# Domain‐general scientific reasoning abilities in kindergarten independently predict the mathematics ability of elementary school children

**DOI:** 10.1111/bjdp.70013

**Published:** 2025-08-20

**Authors:** Christopher Osterhaus, Susanne Koerber

**Affiliations:** ^1^ University of Vechta Vechta Germany; ^2^ Freiburg University of Education Freiburg Germany

**Keywords:** domain‐specific, elementary school, mathematics, scientific reasoning domain‐general

## Abstract

Whether scientific reasoning is a domain‐general or domain‐specific ability remains controversial. This longitudinal study followed 53 German aged 6–9 years (31 females, 22 males) from kindergarten into elementary school to investigate how kindergarten‐age scientific reasoning, intelligence, and disciplinary knowledge influence their third‐grade mathematics and German language abilities (based on teacher ratings). Scientific reasoning was assessed with comprehensive inventories (the Science‐Kindergarten and the Science‐Primary School Reasoning Inventories). Intelligence, language abilities (receptive language and text comprehension in kindergarten and elementary school, respectively), and kindergarten mathematics were assessed with standardized instruments. Kindergarten scientific reasoning predicted third‐grade mathematics abilities independent of parental education levels, and also the intelligence and kindergarten mathematics ability of the children. The language ability of children was predicted solely by kindergarten language abilities. These findings support the view that scientific reasoning is a domain‐general science skill, which is different from intelligence and relevant for mathematics learning among elementary school students.

**Statement of Contribution**

Existing knowledge

- Scientific reasoning is considered a cognitive skill relevant for problem‐solving and learning.
- It is unclear whether scientific reasoning is a domain‐general or domain‐specific skill.
- Prior research links intelligence and early academic skills to later school performance.

New knowledge added

- Kindergarten scientific reasoning independently predicts third‐grade mathematics abilities.
- Kindergarten language abilities—but not scientific reasoning—predict third‐grade language ability.
- Findings support scientific reasoning as a domain‐general science skill important for math learning.

## INTRODUCTION

Scientific reasoning is defined as intentional knowledge‐seeking (Kuhn, 2002) and it comprises diverse abilities in areas, such as experimentation, data interpretation, and understanding of the nature of science (Osterhaus et al., 2020). Scientific reasoning is a 21st‐century skill (Trilling & Fadel, 2009) and a key aspect of early science, technology, engineering, and mathematics (STEM) abilities (Koerber & Osterhaus, 2021). There has recently been considerable debate around whether scientific reasoning is a domain‐general or domain‐specific STEM ability (Fischer et al., 2018). Proponents of the domain‐general view suggest that there is overlap between scientific disciplines (Renkl, 2018), the conceptual core, and the common underpinnings of scientific reasoning (Sodian, 2018), and that domain‐general (or cross‐domain) scientific reasoning skills foster the acquisition of new disciplinary knowledge across science domains by students (Hetmanek et al., 2018). Proponents of the domain‐specific view therefore suggest that each field of science involves independent methods and reasoning styles (Kind & Osborne, 2017), rendering the teaching of broad scientific reasoning skills less relevant than teaching domain or disciplinary knowledge (Samarapungavan, 2018).

The debate on the domain‐general and domain‐specific nature of scientific reasoning is highly relevant for education practice and policy since it informs about the ideal contents and outcomes of science education. However, this has vastly remained theoretical; there is surprisingly little empirical evidence available for resolving this debate.

Studies that have indicated associations between scientific reasoning (or its subaspects) and science knowledge within STEM disciplines such as physics or mathematics (Chinnappan, 2017; Koerber & Osterhaus, 2019, 2021; Schwichow et al., 2020; van der Graaf et al., 2018) have mostly had a cross‐sectional or prospective (as opposed to longitudinal) design, and are therefore not informative regarding the issue of domain generality versus specificity. This is because both approaches predict significant associations: the domain‐general view predicts that children acquire science knowledge through applying their scientific reasoning skills, while the domain‐specific view predicts that children acquire scientific reasoning abilities through acquiring relevant heuristics through science learning (Schauble, 2018).

Studies that have revealed beneficial effects of mature scientific reasoning (experimentation) skills on science knowledge acquisition (Edelsbrunner et al., 2018) are equally uninformative regarding the issue of domain generality versus specificity, since these studies have only shown this association in a single‐domain inquiry learning context (e.g., physics), where students learn by conducting small experiments. Given the relevance of experimentation skills in this type of learning (and its relevance as a reasoning style within physics), it is not surprising that this association—which implies little or no knowledge transfer—occurs.

Deciding between these two views requires longitudinal studies focusing on science domains that are close, but different enough from the *scientific method*. The scientific method is the standard way of conceiving the experimentation or data interpretation skills of science (Engelmann et al., 2018), and it is often used to measure scientific reasoning (Koerber & Osterhaus, 2019; Nyberg et al., 2020; Osterhaus et al., 2020; van de Sande et al., 2019). Domain‐general scientific reasoning skills, which imply a transfer of skills, should be argued through research examining the associations between scientific reasoning (when conceived in the terms of the scientific method) and the transfer of skills between scientific disciplines with scientific reasoning styles that are not experimental, such as mathematics (Kind & Osborne, 2017).

The above‐described situation prompted the present study to investigate the longitudinal associations between scientific reasoning and the mathematics abilities of students, while controlling for prior intelligence and relevant disciplinary knowledge. Specifically, we investigated whether kindergarten scientific reasoning predicts elementary school mathematics abilities independent of general cognitive development (intelligence and language abilities) and relevant kindergarten disciplinary knowledge (i.e., knowledge about the mathematical concepts quantities and numbers). We also investigated associations between kindergarten scientific reasoning and elementary school German language performance, again controlling for general cognitive development and relevant kindergarten disciplinary skills (i.e., receptive language understanding).

If the domain‐general view is correct, then the mathematics abilities of children—but not their abilities in German—should be independently predicted by their prior disciplinary knowledge and their scientific reasoning skills. In contrast, if the domain‐specific view is correct, then kindergarten disciplinary knowledge alone should explain variations in subsequent mathematics abilities. We expected scientific reasoning to be a domain‐general *science* ability, meaning that we hypothesized that it independently predicts later mathematics abilities but not German language ability.

Comprehensive scientific reasoning skills first develop at kindergarten and elementary school ages (Koerber & Osterhaus, 2019, 2021; Osterhaus et al., 2016; Sodian et al., 1991), and children acquire ample disciplinary science knowledge during elementary school, with individual differences between students being highly consistent over time (Kähler et al., 2020; Morgan et al., 2016). This consistency makes elementary schools a suitable or even perfect period to critically evaluate the validity of the domain‐general view, because any effect from scientific reasoning on acquiring disciplinary science knowledge must exceed the substantial influence of previous science knowledge.

To obtain holistic ability estimates of the mathematics and German language abilities of children, we studied teacher ratings (marks). Mathematics teachers are sensitive to student performance when rating their abilities (Timmermans et al., 2021); teacher ratings suggest substantial correlations with standardized test scores (Pedulla et al., 1980). In addition, these correlations are stronger than standardized tests with later academic outcomes (Rimfeld et al., 2019) and are therefore considered more reliable.

## MATERIALS AND METHODS

This longitudinal study followed children from kindergarten (ages 6–7) to third grade (ages 8–9) to examine the predictive role of early scientific reasoning on later academic outcomes and was part of a larger project investigating cognitive development in middle childhood (Osterhaus & Koerber, 2023).

### Participants

The participants were 53 children aged 6–9 years (31 females, 22 males) who were part of a longitudinal study on scientific reasoning development, and whose third‐grade ability scores (teacher ratings, marks) in mathematics and German were obtained. The children were aged 6.09 ± 0.30 and 8.98 ± 0.30 years (mean ± standard deviation) during the first (kindergarten) and second (third grade) waves, respectively. The children were from or close to a midsized university city in Germany. A language other than German was spoken at home by five children (9.6%). Parental informed consent and child assent were obtained from all participants.

### Measures

#### Mathematics and German language abilities (3rd‐grade teacher ratings)

The mathematics and German marks of the children were collected in third grade to obtain an estimate from the teachers of their abilities in these two subjects (see Table 1). In Germany, elementary school marks range from 1 (‘excellent’ highest mark) to 6 (‘not passed’, lowest mark). For clarity, all scores were inverted so that higher values indicate better performance in all analyses and descriptive statistics. The correlations between the two teacher ratings and the correlation between third‐grade German marks and the standardized assessment of text comprehension are listed in Table 2, which suggests the validity of the teacher ratings.

**TABLE 1:** Descriptive statistics for all measures and parental education levels.

	*n*	*M*	SD
Grade 3 (Marks)			
Mathematics (1–6)	53	4.70	1.17
German (1–6)	53	4.53	1.07
Kindergarten			
Mathematics (0–12)	53	7.58	2.18
Intelligence (0–10)	53	7.85	2.31
Language (0–12)	53	5.28	2.00
Scientific reasoning (raw score, 0–30)	53	14.08	4.19
Scientific reasoning (scaled score)	53	−0.31	0.82
Grade 3 (Cognitive)			
Intelligence (0–15)	53	9.34	2.75
Language (text comprehension) (0–20)	53	11.70	6.13
Scientific reasoning (scaled score)	53	1.37	0.86
Parents			
Parental education level (1–5)	50	2.17	1.29
Low‐track school		7 (14%)	
Medium‐track school		19 (38%)	
Advanced vocational training		15 (30%)	
Bachelor's degree		2 (4%)	
Master's degree		7 (14%)	

*Note*: Means and standard deviations are shown for continuous variables; parental education is shown as the number of participants and percentage in each category.

**TABLE 2:** Correlations between all assessed measures.

	Kindergarten				Grade 3 (cognitive)			Grade 3 (Marks)	
	Int.	Lang.	Math	SR	Int.	Lang.	SR	Math	German
Kindergarten									
Intelligence		.220	.404**	.152	.427**	.142	.216	.311*	.299*
Language			.392**	.280*	−.049	.174	.249^a^	.288*	.420**
Mathematics				.245^a^	.146	.044	.350*	.433**	.220
Scientific reasoning					−.114	.075	.544***	.385**	.337*
Grade 3 (Cognitive)									
Intelligence						.006	.022	.134	−.043
Language							.229^a^	.269^a^	.398**
Scientific reasoning								.455**	.211
Grade 3 (Mark)									
Mathematics									.654***
Parental education level	.044	−.116	−.235	.129	−.029	.348*	.211	.203	.253^a^

Abbreviations: Int., intelligence; Lang., language abilities; SR, scientific reasoning.

^a^ *p* < .1.

* *p* < .05.

** *p* < .01.

*** *p* < .01.

#### Knowledge of mathematical concepts (kindergarten)

Children's understanding of quantities and numbers was assessed using 11 items from the Vienna Development Test (Kastner‐Koller & Deimann, 2002) that instruct children to compare quantities and to perform simple arithmetic operations on them. Internal consistency was estimated using Cronbach's α (Cronbach, 1951), a widely used reliability measure in educational and developmental research (Tavakol & Dennick, 2011). For developmental measures, *α* values of .60–.70 are often considered acceptable due to the complexity of constructs in early childhood, and even relatively low (e.g., .50) levels of criterion reliability do not seriously attenuate validity coefficients (Schmitt, 1996). Cronbach's *α* for children's knowledge of mathematical concepts was .64, indicating reliability.

#### Scientific reasoning (kindergarten and 3rd grade)

**FIGURE 1:**
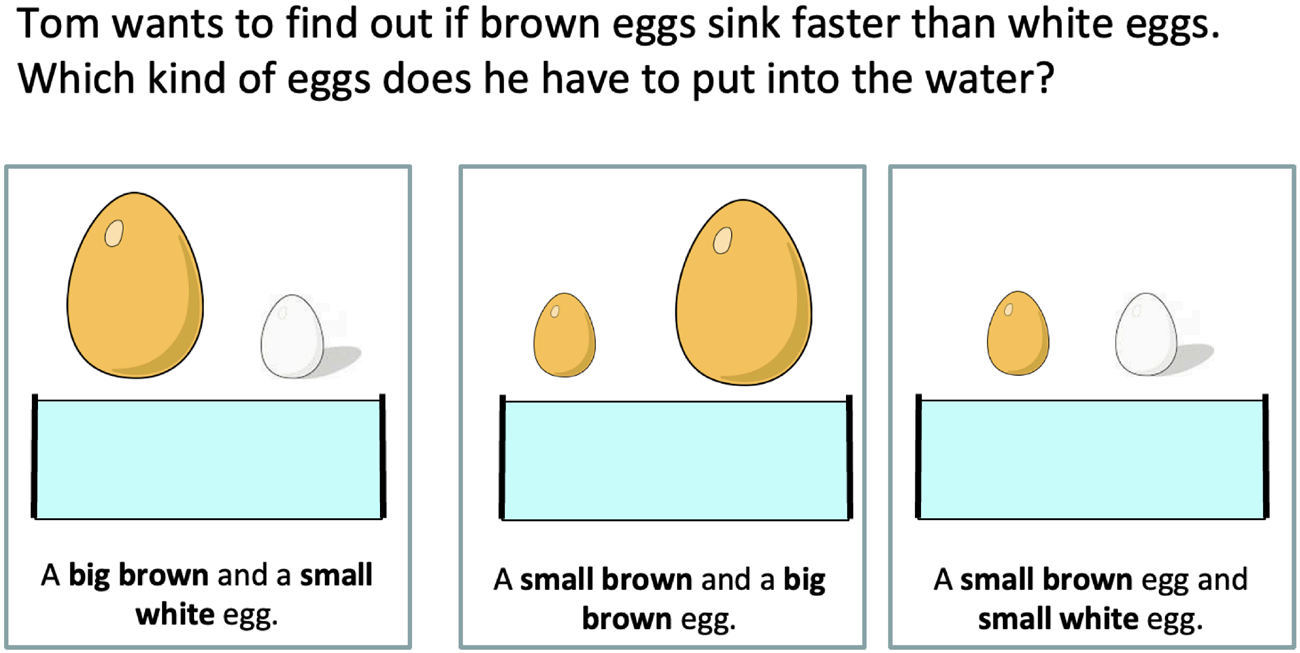
Sample item from the SK‐I assessing children's mastery of the control‐of‐variables strategy (experimentation). Reprinted from Koerber and Osterhaus (2019).

Two closed‐response instruments were used to measure scientific reasoning: the Science‐Kindergarten Inventory (SK‐I) (Koerber & Osterhaus, 2019) and the Science‐Primary School Reasoning Inventory (SPR‐I) (Osterhaus et al., 2020). SK‐I is a 30‐item instrument that assesses emerging experimentation abilities, data interpretation, and the understanding of the nature of science (10 items each) (Figure 1 displays a sample item). All items had three answer options, and three individual interviews (each ~20 min) were conducted, with no corrective feedback given. The five SPR‐I items included in this study assessed more advanced scientific reasoning abilities, including experimentation (two items), data interpretation (one item), and understanding of the nature of science (two items). All five items had three answer options, each representing a distinct level of understanding (Osterhaus et al., 2020).

SK‐I was applied to kindergarten and third‐grade children. In the third grade, the five more difficult items from the SPR‐I were introduced, and half of the SK‐I items were assessed in a whole‐class testing procedure (as opposed to individual interviews) to improve cost‐effectiveness. Responses to the different items were linked based on the responses to the 15 SK‐I anchor items (interviews), and abilities were calculated based on weighted likelihood estimates (WLE). This data analysis was conducted simultaneously from a total of six waves of the larger project (see the scale analysis in Data S1). The reliability of the scientific reasoning scale was good across all waves, with a WLE person separation reliability coefficient of .832.

#### Language

Measures of receptive language understanding from the Intelligence and Developmental Scales (Grob et al., 2009) and text comprehension (Lenhard & Schneider, 2006) were used to obtain estimates of the language abilities of children. The receptive language test presented the children with 12 sentences of increasing grammatical complexity (e.g., ‘the girl who is being chased by the boy is holding the cloth that before was in the cat basket’), which the children were instructed to demonstrate using small toy figures. The children received full credit (1 point) when both the sentence order and content were correct, while they received partial credit (.5 points) when only the content was correct. The measure of text comprehension (Lenhard & Schneider, 2006) presented the children with 20 short texts and comprehension questions (with four answer options). The reliability of the receptive language assessment was acceptable, yielding a Cronbach's *α* of .73; the reliability of the text comprehension measure was excellent, yielding a Cronbach's *α* of .94.

#### Intelligence

Proxies for intelligence were obtained via the 10‐item ‘analogical reasoning’ subtest of the Vienna Development Test (Kastner‐Koller & Deimann, 2002) and the 15‐item ‘progressive matrices’ subtest from the Culture Fair Intelligence Test (CFIT) (Weiß, 2006) for kindergarten and third grade, respectively. The reliabilities of the analogical reasoning test and CFIT subtest were acceptable, with Cronbach's α values of 0.78 and 0.70, respectively.

#### Parental education level

The parental education level was assessed using a sociodemographics questionnaire and coded as follows: (1) low‐track school completed (9 years of education); (1.5) medium‐track school completed (10 years of education); (2) college preparatory education or vocational training completed (12 or 13 years of education); (3) advanced vocational training completed (~15 years of education); (4) bachelor's degree completed; and (5) master's degree completed.

### Procedure

All individual interviews for kindergarten and third‐grade children were conducted by trained researchers on separate days in quiet, independent rooms at their respective kindergartens or schools (teachers were absent during the interviews). Illustrated booklets were used during the whole‐class assessment for third‐grade children; the content and answer options of all tasks were presented in a PowerPoint presentation and read out to the children by an experimenter. To guarantee that children would work quietly and independently, one or two test assistants were present during the whole‐class assessments. The teachers supplied the children's mathematics and German marks; the parents completed sociodemographics questionnaires at home and returned them to the teachers (obtained from 50 families, response rate of 94%).

### Data analysis

We conducted descriptive statistics, Pearson correlations, and hierarchical regression analyses. Dependent variables were teacher‐rated third‐grade mathematics and German language abilities. Independent variables included kindergarten scientific reasoning, intelligence, kindergarten mathematics, and language skills, as well as parental education. Analyses were conducted in IBM SPSS Statistics (version 28).

## RESULTS

### Core abilities in mathematics and German

The mathematics and German marks of third‐grade children were 4.70 ± 1.17 and 4.53 ± 1.07, respectively (range 1–6), with both marks classed ‘good’.

A multivariate analysis of variance (ANOVA) revealed a significant difference between females and males [Wilk's *λ* = .874, *F*(2, 50) = 3.605, *p* = .034, partial *η* ^2^ = .126]. Two follow‐up ANOVAs indicated that females (2.22 ± 1.06) had better German marks than males (2.82 ± 1.01) [*F*(1, 51) = 4.211, *p* = .045, partial *η* ^2^ = .076], while there was no difference for mathematics marks (2.29 ± 1.32 and 2.31 ± .95 for females and males, respectively) [*F*(1, 51) = 0.007, *p* = .933].

### Correlations between mathematics and German language abilities, general cognitive abilities, and scientific reasoning

Table 2 reports the correlations between all of the variables investigated in this study. A substantial correlation emerged between third‐grade mathematics ability and scientific reasoning scores for kindergarten and third grade, and also between kindergarten mathematics abilities and kindergarten intelligence and language abilities. There was a correlated trend between third‐grade mathematics abilities and concurrent language abilities, but no significant correlation between third‐grade mathematics abilities and concurrent intelligence. Partial correlation analysis indicated that the concurrent association between third‐grade mathematics abilities and scientific reasoning was independent of language abilities (*ρ* = .419, *p* = .002). This was the same for the correlation between third‐grade mathematics abilities and kindergarten scientific reasoning (controlling for kindergarten language abilities and intelligence) (*ρ* = .318, *p* = .023).

The third‐grade German language ability was correlated with the concurrent language abilities, and also with language abilities, intelligence, and scientific reasoning in kindergarten; but it had no concurrent association with third‐grade scientific reasoning scores or kindergarten mathematics ability.

Parental education level was correlated with the third‐grade text comprehension (language) measure; there was also a correlating trend with the third‐grade German score.

### Predicting third‐grade mathematics abilities using kindergarten indicators

Hierarchical regression analysis with third‐grade mathematics grades as the dependent variable (Table 3) indicated that the model with the greatest amount of explained variance was a model including predictions of kindergarten mathematics abilities and scientific reasoning, when intelligence was controlled for (model 2d). It must be considered that the kindergarten intelligence prediction, which was significant in an initial model (model 2a), was not significant when kindergarten mathematics was included in the model (model 2c). Kindergarten scientific reasoning exerted a significant effect, even when kindergarten mathematics was considered. The effects of kindergarten mathematics and scientific reasoning were comparable in magnitude.

**TABLE 3:** Results of a hierarchical regression analysis: predicting mathematics and German language grades in third grade.

	*β*	*t*	*p*	Change in *F*	Corrected *R* ^2^
*DV, Mathematics grade in third‐grade*					
Model 1				*F*(1, 48) = 2.071	.021
Parents' Education	.203	1.439	.157		
Model 2a				*F*(1, 51) = 5.443*	.079
Intelligence (K)	.311	2.333*	.024		
Model 2b				*F*(1, 50) = 2.983^a^	.113
Intelligence (K)	.260	1.939^a^	.058		
Language (K)	.231	1.727^a^	.090		
Model 2c				*F*(1, 50) = 7.132*	.178
Intelligence (K)	.162	1.179	.244		
Mathematics (K)	.367	2.671^a^	.010		
Model 2d				*F*(1, 49) = 5.345*	.243
Intelligence (K)	.144	1.088	.282		
Mathematics (K)	.304	2.257*	.028		
Scientific reasoning (K)	.288	2.312*	.025		
*DV, German Grade in third grade*					
Model 3				*F*(1, 48) = 3.272^a^	.044
Parents' education	.253	1.809^a^	.077		
Model 4a				*F*(1, 51) = 4.993*	.071
Intelligence (K)	.299	2.234*	.030		
Model 4b				*F*(1, 50) = 8.490**	.190
Intelligence (K)	.216	1.692*	.097		
Language (K)	.373	2.914**	.005		
Model 4c				*F*(1, 49) = 2.923	.220
Intelligence (K)	.196	1.551	.127		
Language (K)	.316	2.343*	.019		
Scientific reasoning (K)	.219	1.710^a^	.094		

^a^ *p* < .1.

* *p* < .05.

** *p* < .01.

Hierarchical regression analysis with third‐grade German language ability as the dependent variable indicated that predictions were only significant in kindergarten language abilities and intelligence, but not in kindergarten scientific reasoning or mathematics.

## DISCUSSION

There has been considerable debate about whether scientific reasoning is a domain‐general or domain‐specific skill (Fischer et al., 2018). The domain‐general view suggests that scientific reasoning is a relevant skill across STEM disciplines, and helps students to acquire science knowledge. In contrast, the domain‐specific view suggests that disciplinary STEM knowledge involves highly specific reasoning styles, meaning that scientific reasoning does not have any effect on the knowledge acquisition of students in STEM that exceeds the influence of prior disciplinary knowledge.

The present study addressed this debate by examining whether early scientific reasoning predicts later academic performance. Our findings show that scientific reasoning in kindergarten significantly predicts third‐grade mathematics ability, even after controlling for intelligence and prior mathematics skills, but does not predict German language ability. This pattern of results supports the STEM domain‐general view, indicating that early individual differences in scientific reasoning are relevant for children's knowledge acquisition in STEM disciplines. It is worth noting that no comparable effect was found for German language ability, suggesting that scientific reasoning is not a general cognitive skill like intelligence (see also Koerber et al., 2015), but rather a domain‐general science skill.

The effects of scientific reasoning and mathematics abilities in kindergarten on third‐grade mathematics marks were comparable in magnitude, suggesting that prior individual differences in these two aspects are equally relevant for successful STEM learning among elementary school children. These findings align with prior cross‐sectional evidence linking scientific reasoning to STEM skills (Chinnappan, 2017; Koerber & Osterhaus, 2019, 2021; Schwichow et al., 2020; van der Graaf et al., 2018). However, to our knowledge, no previous longitudinal studies have examined this relation; making our findings novel in demonstrating predictive validity over a multi‐year span.

The total explained variance in third‐grade mathematics performances (based on kindergarten predictors) was as high as 24%, which underlines the importance of effective early STEM teaching, and would ideally begin in kindergarten. The need for early interventions to foster the science skills of children has been increasingly recognized, and there is a growing interest in researching the scientific competencies of young children (Fridman et al., 2020; Studhalter et al., 2021).

The differing patterns of results between mathematics and German indicate that scientific reasoning is relevant for the natural sciences, but less so for the humanities. It is worth noting that the scientific reasoning measure was conceived while considering the scientific method, focusing on experimentation, data interpretation, and an understanding of the nature of science. It appears reasonable to assume that stronger associations between scientific reasoning and German language ability may have emerged if we had employed more language‐based scientific reasoning measures, such as those assessing argumentation skills (Iordanou et al., 2019).

The effect of kindergarten domain‐general intelligence became insignificant during the hierarchical regression analysis when the specific disciplinary knowledge predictors were included. The effect was not significant for mathematics abilities when kindergarten mathematics knowledge was included, or for German when the kindergarten language score was included. This supports the view of a strong effect of prior knowledge on learning and domain specificity on reasoning, exceeding the effects of a domain‐general intelligence skill.

Despite intelligence not being a significant predictive factor, scientific reasoning remained a significant predictor even when considering prior disciplinary knowledge. This supports the view that scientific reasoning is a general science ability, which is relevant for mathematics knowledge acquisition among elementary school students. This finding is consistent with prior suggestions of significant associations between scientific reasoning and physics and mathematics knowledge (Koerber & Osterhaus, 2019; Schlatter et al., 2020). Alongside the high stability of scientific reasoning (i.e., a coefficient of .54 for the correlation between kindergarten and third grade), this finding suggests that scientific reasoning is indeed a domain‐general science ability with consistent individual differences that appear to be less affected by the specific scientific experiences of elementary school children.

Because individual differences among science knowledge are stable during kindergarten and elementary school (Kähler et al., 2020; Morgan et al., 2016), elementary school age is an important period for investigating the relative influences of scientific reasoning and prior science disciplinary knowledge, as effects need to be substantial to be realized. However, our choice to study the domain generality versus specificity issue at elementary school ages restricts the conclusions of the present study to this age group, and domain‐specific influences of the students' ability in mathematics may increase in later grades (Geary et al., 2017). As students get older, they acquire more science knowledge through schooling. As this knowledge increases and becomes more specific, the students also become acquainted with the specific reasoning styles that may be prominent across different disciplines. Future research should therefore address the domain generality versus specificity issue by analysing older student populations.

Kindergarten‐age scientific reasoning had a substantial effect on third‐grade mathematics ability; alongside prior mathematics knowledge, it explained almost 25% of the variance. This underlines the importance of fostering scientific reasoning early, since it is a 21st‐century skill. While a strength of this study was the long interval between the two measurements (kindergarten to third grade), a potential limitation is the relatively small sample. All of the participants in this study had also participated in a larger project on cognitive development in middle childhood; however, teacher ratings (marks) were only obtained for one of the cohorts. Additionally, while teacher ratings have been suggested as reliable estimates of children's abilities (Rimfeld et al., 2019), future research could validate our conclusions by also including standardized measures of elementary school mathematics ability. However, the significant correlation that the present study found between the German language ability of children and a standardized assessment of text comprehension supports the view that the teacher ratings in the present study were valid.

## CONCLUSION

Kindergarten scientific reasoning significantly predicts third‐grade mathematics abilities, independent of the effects from kindergarten intelligence and prior disciplinary knowledge. This finding supports the view that scientific reasoning is a domain‐general science skill that differs from general intelligence and is relevant for elementary school science learning.

## AUTHOR CONTRIBUTIONS

**Christopher Osterhaus:** Conceptualization; methodology; data curation; investigation; formal analysis; project administration; writing – original draft; writing – review and editing. **Susanne Koerber:** Conceptualization; investigation; methodology; data curation; writing – review and editing; funding acquisition; project administration.

## CONFLICT OF INTEREST STATEMENT

The authors declare no conflict of interest.

## Supporting information


Data S1:

## Data Availability

The data that support the findings of this study are available on request from the corresponding author. The data are not publicly available due to privacy or ethical restrictions.
