# Supplementary material for: Domain‐general scientific reasoning abilities in kindergarten independently predict the mathematics ability of elementary school children
Source: Br J Dev Psychol. 2025 Aug 20;44(1):134–45. doi: 10.1111/bjdp.70013 (PMC12884364; doi:10.1111/bjdp.70013)
Supplement: Supplementary file 1 — Data S1: [file BJDP-44-134-s001.docx]

Domain-general scientific reasoning abilities in kindergarten independently predict the mathematics ability of elementary-school children

Online Supplement

**Results Scale Analysis of Children’s Scientific Reasoning Performance**

Rasch analysis was used to estimate the children’s scientific-reasoning abilities. Estimates of 3^rd^-grade abilities in mathematics were obtained from only one of the two cohorts participating in the larger project (see Methods); data from both cohorts (*N* = 161) were used for the scientific-reasoning scale analysis, including data from Wave 5 in 4^th^ grade.

Rasch scale analysis provides estimates of the abilities of individuals and item difficulties, thereby allowing ability comparisons across developmentally sensitive item sets with diverse item difficulties. Our small sample did not allow multidimensional longitudinal Rasch modeling (where each wave is modeled on a distinct latent dimension), and so we fitted a single unidimensional Rasch model to the data from all six waves, yielding estimates of ability based on WLE that were used for further analysis. The analysis was conducted using the eRm package (Mair & Hatzinger, 2007).

Five children whose responses did not vary and who responded incorrectly to all items were excluded from the analysis. The fit of the Rasch model was evaluated using chi-square fit statistics, in particular the information-weighted *infit* mean-squares (MNSQ) statistic. The infit MNSQ is based on the sum of squared standardized residuals weighted by the individual variance. This reduces the impact of unexpected responses far from the item, which makes this statistic less sensitive than the unweighted *outfit* MNSQ to unexpected responses by children for whom the item is far too easy or too difficult. Because of the wide age range and the broad range of abilities and item difficulties in our study, the item fit under the Rasch model was evaluated using the infit MNSQ, with cutoff values of 1±0.2 to identify misfit (Smith et al., 1998).

The unidimensional Rasch model showed a good fit to the 50 scientific reasoning items (30 SK-I interview, 15 SK-I paper-and-pencil, and 5 SPR-I items), as indicated by the infit statistics (Table S-1). Eight items were identified as misfitting and removed from the analysis: two, one, and five of these items measured the nature of science understanding, experimentation, and data interpretation, respectively. The misfit of the four data-interpretation items (assessing interpretations of covariation data) is consistent with prior findings (Koerber & Osterhaus, 2019), and suggests that these early-emerging abilities are not suitable indicators of genuine scientific-reasoning abilities in older children (Kuhn & Pearsall, 2000). No other items were excluded; the remaining 42 items revealed good reliability (WLE person-separation reliability = .832).

There was a good match between item difficulties and the abilities of individuals, with no particular clustering of items (see Figure S-1), and easy and difficult items being widely dispersed across all components and also the interview and paper-and-pencil methods. As expected, four of the five most-difficult items came from the advanced SPR-I.

**References**

Koerber, S., & Osterhaus, C. (2019). Individual differences in early scientific thinking: Assessment, cognitive influences, and their relevance for science learning. *Journal of Cognition and Development*, 1-24. <https://doi.org/10.1080/15248372.2019.1620232>

Kuhn, D., & Pearsall, S. (2000). Developmental origins of scientific thinking. *Journal of Cognition and Development*, *1*(1), 113-129.  [https://doi.org/10.1207/S15327647JCD0101N_11](https://psycnet.apa.org/doi/10.1207/S15327647JCD0101N_11)

Mair, P., & Hatzinger, R. (2007). Extended Rasch modeling: The eRm package for the application of IRT models in R. *Journal of Statistical Software*, *20*(9), 1-20. <http://www.jstatsoft.org/v20/i09/>

Smith, R. M., Schumacker, R. E., & Bush, M. J. (1998). Using item mean squares to evaluate fit to the Rasch model. *Journal of Outcome Measurement*, *2*(1), 66-78.

Table S-1.

Results of the Rasch analysis.

|  |  |  |  | Ability on SK-I (%) and SPR-I (0-2) | | | | | |  | MNSQ | |
| --- | --- | --- | --- | --- | --- | --- | --- | --- | --- | --- | --- | --- |
| Item | Test | Method | Aspect | K0 | K1 | G1 | G2 | G3 | G4 | Diff. | *Outfit* | *Infit* |
| Exp.01 | SK-I | Int | CT | 50.85 | 52.83 | 64.57 | 71.55 |  |  | -0.388 | 1.103 | 1.058 |
|  | SK-I | PP | CT |  |  |  |  | 95.54 | 97.44 | -2.081 | 0.423 | 0.849 |
| Exp.02 | SK-I | Int | CT | 55.93 | 54.72 | 68.50 | 75.00 | 86.07 | 95.80 | -0.654 | 0.766 | 0.909 |
| Exp.03 | SK-I | Int | CT | 15.25 | 17.61 | 29.92 | 48.70 |  |  | 1.166 | 1.004 | 0.975 |
|  | SK-I | PP | CT |  |  |  |  | 76.79 | 92.32 | -0.417 | 0.672 | 0.810 |
| Exp.04 | SK-I | Int | CT | 11.86 | 28.30 | 34.65 | 47.41 | 50.00 | 58.82 | 1.052 | 0.854 | 0.903 |
| Exp.05 | SK-I | Int | CVS | 38.98 | 45.28 | 55.91 | 62.93 |  |  | 0.016 | 1.016 | 1.013 |
|  | SK-I | PP | CVS |  |  |  |  | 93.75 | 96.58 | -1.742 | 0.519 | 0.840 |
| Exp.06 | SK-I | Int | CVS | 44.07 | 46.54 | 61.42 | 61.21 | 86.89 | 93.28 | -0.267 | 0.740 | 0.850 |
| Exp.07 | SK-I | Int | CVS | 44.07 | 51.57 | 69.29 | 75.00 |  |  | -0.441 | 0.790 | 0.813 |
|  | SK-I | PP | CVS |  |  |  |  | 99.11 | 98.29 | -3.095 | 1.335 | 0.893 |
| Exp.08 | SK-I | Int | CVS | 18.64 | 30.82 | 58.27 | 65.52 | 86.89 | 96.64 | -0.003 | 0.657 | **0.723** |
| Exp.09 | SK-I | Int | CVS | 30.51 | 58.49 | 74.02 | 71.55 |  |  | -0.495 | 0.752 | 0.820 |
|  | SK-I | PP | CVS |  |  |  |  | 97.32 | 99.15 | -2.801 | 0.370 | 0.900 |
| Exp.10 | SK-I | Int | CVS | 44.07 | 52.83 | 69.29 | 71.55 | 93.44 | 95.80 | -0.619 | 0.697 | 0.832 |
| Exp.A07 | SPR-I | PP | CVS |  |  |  |  | 0.974 | 1.096 | 2.102 | 1.468 | 1.106 |
| Exp.A08 | SPR-I | PP | CT |  |  |  |  | 1.171 | 1.345 | 2.017 | 1.094 | 0.979 |
|  |  |  |  |  |  |  |  |  |  |  |  |  |
| Data.01 | SK-I | Int | PC | 62.71 | 76.73 | 85.04 | 86.32 | 91.80 | 49.58 | -0.883 | 1.797 | **1.252** |
| Data.02 | SK-I | Int | IC | 52.54 | 52.20 | 45.52 | 37.61 | 44.26 | 45.00 | 0.698 | 1.586 | **1.421** |
| Data.03 | SK-I | Int | NC | 52.54 | 74.21 | 90.55 | 92.31 | 90.98 | 96.67 | -1.519 | 0.912 | 0.926 |
| Data.04 | SK-I | Int | CD | 8.47 | 22.01 | 28.35 | 30.77 | 35.25 | 50.00 | 1.572 | 1.250 | 1.115 |
| Data.05 | SK-I | Int | CD | 18.64 | 27.67 | 34.65 | 47.86 | 40.16 | 48.33 | 1.166 | 1.309 | **1.214** |
| Data.06 | SK-I | Int | CD | 25.42 | 38.99 | 48.82 | 59.83 |  |  | 0.327 | 1.135 | 1.100 |
|  | SK-I | PP |  |  |  |  |  | 67.86 | 79.49 | 0.308 | 0.998 | 1.025 |
| Data.07 | SK-I | Int | PC | 55.93 | 51.57 | 52.76 | 42.74 |  |  | 0.113 | 1.468 | **1.330** |
|  | SK-I | PP | PC |  |  |  |  | 69.64 | 61.54 | 0.736 | 1.169 | 1.140 |
| Data.08 | SK-I | Int | PC | 55.93 | 79.87 | 80.31 | 83.76 |  |  | -1.367 | 1.038 | 1.013 |
|  | SK-I | PP | PC |  |  |  |  | 87.50 | 88.03 | -0.689 | 0.918 | 0.931 |
| Data.09 | SK-I | Int | IC | 45.76 | 57.86 | 33.07 | 35.04 |  |  | 0.398 | 1.495 | **1.346** |
|  | SK-I | PP | IC |  |  |  |  | 33.93 | 34.19 | 2.148 | 1.124 | 1.036 |
| Data.10 | SK-I | Int | NC | 52.24 | 71.70 | 81.10 | 91.45 |  |  | -1.310 | 1.007 | 1.011 |
|  | SK-I | PP | NC |  |  |  |  | 95.54 | 97.44 | -2.081 | 0.472 | 0.869 |
| Data.A06 | SPR-I | Int | CD |  |  |  |  | 0.992 | 0.871 | 2.348 | 1.119 | 0.988 |

| Item | Test | Method | Aspect | K0 | K1 | G1 | G2 | G3 | G4 | Diff. | *Outfit* | *Infit* |
| --- | --- | --- | --- | --- | --- | --- | --- | --- | --- | --- | --- | --- |
| NoS.01 | SK-I | Int | Do | 30.51 | 40.25 | 63.78 | 66.38 |  |  | 0.006 | 1.018 | 1.030 |
|  | SK-I | PP | Do |  |  |  |  | 81.25 | 82.91 | -0.215 | 0.679 | 0.869 |
| NoS.02 | SK-I | Int | Do | 30.51 | 48.43 | 72.44 | 68.97 |  |  | -0.272 | 0.903 | 0.920 |
|  | SK-I | PP | Do |  |  |  |  | 99.54 | 98.29 | -2.220 | 0.651 | 0.886 |
| NoS.03 | SK-I | Int | Do | 8.47 | 20.89 | 30.71 | 26.72 |  |  | 1.462 | 0.952 | 1.011 |
|  | SK-I | PP | Do |  |  |  |  | 65.18 | 76.92 | 0.450 | 1.038 | 0.992 |
| NoS.04 | SK-I | Int | Do | 50.85 | 71.70 | 88.98 | 79.31 | 100.0 | 98.32 | -1.419 | 0.508 | **0.774** |
| NoS.05 | SK-I | Int | Do | 23.73 | 38.36 | 56.69 | 56.90 | 77.87 | 85.71 | 0.150 | 0.792 | 0.851 |
| NoS.06 | SK-I | Int | Ask | 10.17 | 9.43 | 23.62 | 30.17 | 42.62 | 53.78 | 1.658 | 1.101 | 1.014 |
| NoS.07 | SK-I | Int | Ask | 20.34 | 35.22 | 49.61 | 50.00 |  |  | 0.527 | 0.879 | 0.898 |
|  | SK-I | PP | Ask |  |  |  |  | 86.61 | 84.62 | -0.490 | 1.100 | 1.018 |
| NoS.08 | SK-I | Int | Ask | 20.34 | 42.14 | 47.24 | 56.90 |  |  | 0.362 | 0.837 | 0.856 |
|  | SK-I | PP | Ask |  |  |  |  | 91.96 | 94.87 | -1.404 | 0.810 | 0.942 |
| NoS.09 | SK-I | Int | Ask | 11.86 | 27.67 | 41.60 | 43.97 | 66.39 | 81.51 | 0.693 | 0.845 | 0.845 |
| NoS.10 | SK-I | Int | Ask | 16.95 | 26.42 | 43.31 | 56.03 | 78.69 | 89.08 | 0.406 | 0.745 | **0.794** |
| NoS.A01 | SPR-I | PP | Do |  |  |  |  | 0.638 | 0.466 | 4.118 | 1.720 | 0.961 |
| NoS.A21 | SPR-I | PP | Do |  |  |  |  | 1.446 | 1.558 | 0.871 | 1.012 | 1.102 |

*Notes*. Diff. = difficulty; MNSQ = mean-squares statistic. Items with the infit MNSQ in boldface were removed due to misfit, as identified by values larger or smaller than 1 ± -.2.

Figure S-1. Wright map showing person abilities and item difficulties.
